# Adjunctive Fasudil Use and Outcomes After Clazosentan Treatment for Aneurysmal Subarachnoid Hemorrhage: A Multicenter DCI Japan Registry Study

**DOI:** 10.3390/jcm15166424

**Published:** 2026-08-19

**Authors:** Shingo Matsuda, Masahito Katsuki, Yusuke Inoue, Toshikazu Hidaka, Yasuhiko Matsumori, Daizo Ishii, Katsumi Takizawa, Yoji Tanaka, Masaki Chin, Motohiro Morioka, Hiroki Kurita, Akihiro Hirayama, Koreaki Irie, Ichiro Nakahara, Nobutaka Horie, Fusao Ikawa

**Affiliations:** 1 Department of Neurosurgery, Graduate School of Biomedical and Health Sciences, Hiroshima University, Hiroshima 734-8551, Hiroshima, Japan; shima2da@hiroshima-u.ac.jp (S.M.); daiishii@hiroshima-u.ac.jp (D.I.); horie@hiroshima-u.ac.jp (N.H.); 2 Department of Neurosurgery, Shimane Prefectural Central Hospital, Izumo 693-8555, Shimane, Japan; yusukeinoue0610@outlook.jp (Y.I.); hidaka@spch.izumo.shimane.jp (T.H.); 3 Physical Education and Health Center, Nagaoka University of Technology, Nagaoka 940-2137, Niigata, Japan; ktk1122nigt@gmail.com; 4 Insight Research Ireland Centre for Data Analytics, School of Health and Human Performance, Dublin City University, D09 YT18 Dublin, Ireland; 5 Sendai Headache and Neurology Clinic, Sendai 982-0014, Miyagi, Japan; ma2mori@gmail.com; 6 Department of Neurosurgery, Japanese Red Cross Asahikawa Hospital, Asahikawa 070-0061, Hokkaido, Japan; ktaki0820ktaki@gmail.com; 7 Department of Neurosurgery, Kyorin University, Mitaka 181-8611, Tokyo, Japan; yoji-tanaka@ks.kyorin-u.ac.jp; 8 Department of Neurosurgery, Kurashiki Central Hospital, Kurashiki 710-0052, Okayama, Japan; mc13552@kchnet.or.jp; 9 Department of Neurosurgery, Kurume University School of Medicine, Kurume 830-0011, Fukuoka, Japan; mmorioka@med.kurume-u.ac.jp; 10 Department of Cerebrovascular Surgery, Saitama Medical University International Medical Center, Hidaka 350-1298, Saitama, Japan; hkurita@saitama-med.ac.jp; 11 Department of Neurosurgery, Tokai University, Isehara 259-1193, Kanagawa, Japan; a-hira@tokai.ac.jp; 12 Department of Neurosurgery, Japan Red Cross Medical Center, Shibuya-ku 150-8935, Tokyo, Japan; irie_koreaki@med.jrc.or.jp; 13 Department of Neurosurgery, Fukuoka Shin Mizumaki Hospital, Mizumaki 807-0051, Fukuoka, Japan; ichiro@mub.biglobe.ne.jp

**Keywords:** adverse events, cerebral vasospasm, clazosentan, fasudil hydrochloride hydrate, subarachnoid hemorrhage

## Abstract

**Background/Objectives**: Subarachnoid hemorrhage (SAH) is frequently complicated by cerebral vasospasm (VS). Clazosentan reduces VS, and fasudil hydrochloride hydrate (Fasudil) is widely used for VS prevention in Japan. However, the benefit of adding Fasudil to clazosentan remains unclear. We investigated whether adjunctive Fasudil was associated with VS-related and functional outcomes in clazosentan-treated aneurysmal SAH. **Methods**: We retrospectively analyzed data from the multicenter “Database of Cohort Study for Outcome of SAH in Japan,” collected from 2020 to 2024. Patients with aneurysmal SAH who underwent surgical clipping or endovascular coiling within 4 days of onset and completed 14 days of clazosentan treatment were included. Outcomes were compared between clazosentan plus Fasudil and clazosentan alone. Multivariable logistic regression assessed factors associated with angiographic vasospasm (AVS), cerebral infarction, and poor functional outcome (modified Rankin Scale 3–6) at discharge and 6 months. **Results**: Among 341 patients, 100 (29.3%) received adjunctive Fasudil and 241 (70.7%) clazosentan alone. AVS occurred in 58/329 (17.6%), cerebral infarction in 58/327 (17.7%), poor functional outcome at discharge in 142/341 (41.6%), and poor functional outcome at 6 months in 82/325 (25.2%). Adjunctive Fasudil was independently associated with higher odds of AVS (adjusted odds ratio [aOR] 2.41, 95% confidence interval [CI] 1.21–4.81), cerebral infarction (aOR 2.10, 95% CI 1.08–4.09), and poor 6-month outcome (aOR 2.88, 95% CI 1.08–7.71), but not with poor functional outcome at discharge (aOR 1.37, 95% CI 0.62–3.03). **Conclusions**: In clazosentan-treated aneurysmal SAH, adjunctive Fasudil use was not associated with additional benefit and was associated with higher odds of AVS, cerebral infarction, and poor functional outcome at 6 months.

## 1. Introduction

Subarachnoid hemorrhage (SAH) is a severe neurological disorder associated with high morbidity and mortality [1,2]. Cerebral vasospasm (VS), a major complication of SAH, contributes to delayed cerebral ischemia (DCI) and poor clinical outcomes [3]. VS develops in 30–70% of SAH cases and results in stroke or death in 15–20% [4]. Although the pathogenesis of DCI is multifactorial [4], prevention of VS remains an important therapeutic goal. Current pharmacologic strategies include nimodipine, a calcium-channel blocker widely used in Western countries [5], and fasudil hydrochloride hydrate (Fasudil), a Rho kinase inhibitor commonly prescribed in Japan [6].

Clazosentan, a selective endothelin A receptor antagonist, inhibits endothelin-1–mediated vasoconstriction. While the CONSCIOUS trials demonstrated its ability to reduce VS, they showed limited impact on functional outcome and mortality [7,8,9]. Similarly, the REACT trial [10] did not demonstrate a significant reduction in DCI. In contrast, a phase 3 randomized controlled trial (RCT) conducted in Japan [11] reported that clazosentan reduced VS and improved 12-week clinical outcomes. Since clazosentan became available for clinical use in Japan in January 2022, these favorable findings have been further supported by several retrospective real-world studies [12,13,14,15,16,17,18,19,20,21,22,23,24,25,26,27,28,29].

In Japan, both clazosentan and Fasudil are approved pharmacological options for the prevention of VS after aneurysmal subarachnoid hemorrhage (aSAH) [30]. Given their distinct pharmacological targets, clazosentan for endothelin-1-mediated vasoconstriction and Fasudil for Rho kinase-mediated pathways, concomitant administration theoretically offers additive protection against VS. In real-world practice, Fasudil is sometimes administered as an adjunct to clazosentan during the VS phase [13,16,18,25,28]. However, this combination was not evaluated in the Japanese phase 3 RCT, which excluded combination prophylactic therapies during the VS phase [11]. Therefore, the clinical value of adjunctive Fasudil to clazosentan remains unclear.

On the other hand, concomitant vasodilatory therapy may also increase the risk of fluid retention and related systemic complications, which could theoretically offset its beneficial effects on cerebral circulation [13,18,28]. Moreover, because adjunctive Fasudil is administered at the treating physician’s discretion in routine clinical practice [31], patients perceived to be at higher risk of vasospasm-related complications may preferentially receive combination therapy, potentially resulting in confounding by indication.

Accordingly, this multi-center retrospective observational study aimed to clarify whether adjunctive Fasudil use is associated with better or worse VS-related and functional outcomes in patients with aSAH treated with clazosentan. We compared angiographic VS (AVS), cerebral infarction, and functional outcomes at discharge and at 6 months between patients receiving clazosentan plus Fasudil and those receiving clazosentan alone. We also performed multivariable analyses to examine the independent association of adjunctive Fasudil use with these outcomes.

## 2. Materials and Methods

### 2.1. Ethical Considerations

The institutional review boards approved this multi-center study of all participating centers; the representative ethical approval was obtained from the Shimane Prefectural Central Hospital Ethics Committee (Approval No. R22-020). Informed consent was waived under an opt-out policy. The study was conducted in accordance with the Declaration of Helsinki and the STROBE guidelines.

### 2.2. Study Design

This was a retrospective observational analysis of data collected prospectively in the multicenter registry.

### 2.3. Dataset Information

This study used data from the “Database of Cohort Study for Outcome of SAH In Japan (DCI Japan),” a multi-center prospective registry of ten high-volume cerebrovascular centers in Japan [29]. Details of the study protocol of DCI Japan are provided in Supplementary File S1. Baseline characteristics, treatment details, VS-related variables, and functional outcomes were prospectively recorded according to the registry protocol. The present research question, patient selection, and statistical analyses were defined and conducted retrospectively.

### 2.4. Consistent Treatment Protocol of General Management for Aneurysmal SAH

General management of aSAH followed the Japanese Guidelines for the Management of Stroke 2021 [32] and local institutional protocols. Details are provided in Supplementary File S2.

### 2.5. Inclusion and Exclusion Criteria

**Figure 1:**
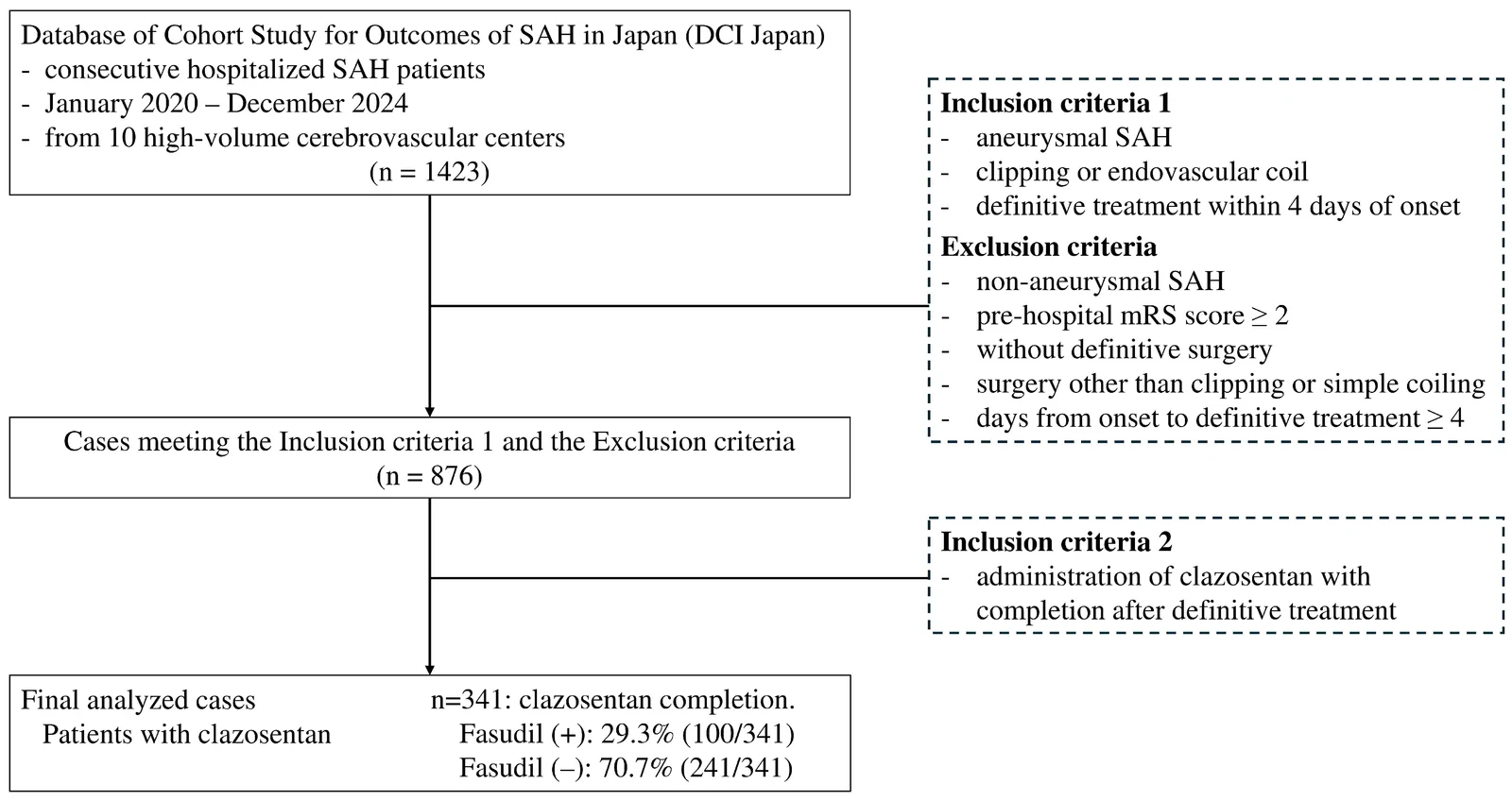
Flow diagram showing patient selection and exclusion criteria for the present study. Of the 1423 patients enrolled in the DCI Japan (Database of Cohort Study for Outcome of SAH in Japan) registry, 341 patients who completed treatment with clazosentan were included in the final analysis. Abbreviations: mRS, modified Rankin Scale; SAH, subarachnoid hemorrhage.

A flow diagram of patient selection is shown in Figure 1. This study included patients with aSAH who underwent surgical clipping or endovascular coil embolization for a ruptured aneurysm within 4 days of symptom onset. Exclusion criteria were: failure to identify a ruptured aneurysm on imaging; non-saccular aneurysms (e.g., dissecting, fusiform, infectious, or traumatic); prehospital modified Rankin Scale (mRS) ≥ 2 or unavailable; absence of definitive treatment; treatments other than simple surgical clipping or coiling (e.g., wrapping, trap and bypass, parent artery occlusion, decompression, or stent-assisted coiling); complex or crossover treatments; missing or inaccurate onset or treatment dates; or treatment initiated ≥4 days after onset. Among patients meeting these criteria, those who received clazosentan and completed 14 days of treatment after definitive treatment were included in the final analysis. Patients who discontinued clazosentan before day 14 were excluded because the present study was designed to evaluate outcomes associated with adjunctive Fasudil during a completed course of clazosentan prophylaxis (Supplementary Table S1).

### 2.6. Definition of AVS and Cerebral Infarction

AVS and cerebral infarction were assessed using computed tomography (CT) angiography or digital subtraction angiography according to local protocols, typically immediately after surgery, on postoperative days 1, 7, and 14, and when new neurological deficits emerged.

AVS was defined as ≥50% narrowing in any cerebral artery on angiography compared with preoperative baseline, excluding changes caused by atherosclerosis, catheter-induced spasm, or vessel hypoplasia. Diagnosis was based solely on imaging [33].

Cerebral infarction was defined as new infarcts occurring within 6 weeks of SAH on CT, magnetic resonance imaging, or autopsy, which were absent on early postoperative imaging and not attributable to surgery, catheters, or hematoma. Infarcts from non-VS mechanisms (e.g., cardioembolism, atherothrombosis) were also included [33,34,35,36].

### 2.7. Management of VS

Before the approval of clazosentan in Japan (January 2022), VS prophylaxis was performed with intravenous Fasudil (90 mg/day for 14 days) [32]. Fasudil is considered one of the standard pharmacologic options for the prevention of VS in Japan and has been widely used in routine clinical practice. After approval, clazosentan (10 mg/h for 14 days) was administered within 24 h of definitive aneurysm treatment as VS prophylaxis in eligible patients, and clazosentan was sometimes administered concomitantly with Fasudil or other prophylaxis in real-world practice [31]. Clazosentan was administered based on clinical availability and physician discretion [31], and was not used reactively for established VS. The decision to administer adjunctive Fasudil in addition to clazosentan was made at the discretion of the treating physician and was not randomized. Because treatment allocation was not randomized, residual confounding cannot be completely eliminated despite multivariable adjustment and propensity score weighting.

Additional therapies, including cilostazol, statins (rosuvastatin, atorvastatin, pitavastatin), and intravenous nicardipine, were administered as clinically indicated. The use of these prophylactic drugs during the VS phase was defined as treatment initiated the day after surgery and continued for 14 days.

### 2.8. Variables and Outcomes

Collected data included age, sex, history of hypertension, diabetes mellitus, stroke, World Federation of Neurosurgical Societies (WFNS) Grade, aneurysm size and location, Fisher CT group, treatment modality (surgical clipping or endovascular coiling), periprocedural management, and VS prophylaxis during the VS phase, including spinal/ventricular/cisternal drains, clazosentan completion, concomitant use of Fasudil, cilostazol, statins, and nicardipine. Radiological interventions for VS included intra-arterial Fasudil and angioplasty. Cerebral complications (hemorrhage, edema, infarction, infection) and systemic complications (infections requiring antibiotics, syndrome of inappropriate secretion of antidiuretic hormone, lung edema, arrhythmia) were documented.

The primary outcome was the occurrence of AVS. Secondary outcomes included cerebral infarction, good (mRS 0–2) or poor (mRS 3–6) functional outcome at discharge, and those at 6 months. We compared these outcomes between patients treated with clazosentan plus Fasudil and those treated with clazosentan alone. These associations were evaluated using the multivariable analyses described below.

### 2.9. Statistical Analyses

Continuous variables are presented as means (standard deviation, SD) or medians [interquartile range (IQR); 25th–75th percentile (Q1–Q3)], and categorical variables are presented as counts and percentages. Group comparisons were performed using *t*-tests, Mann–Whitney U tests, chi-square tests, or Fisher’s exact tests, as appropriate. Variables with *p* < 0.05 in the univariable analysis, together with age, sex, Fisher CT group, and use of adjunctive Fasudil were included in the multivariable logistic regression models. Missing values were handled using ten imputations, and pooled results were reported as the main analyses.

Complete-case analyses were performed as sensitivity analyses and are presented in Supplementary Tables S2–S6. In addition, a sensitivity analysis using stabilized inverse probability of treatment weighting was performed to address confounding by indication, presented in Supplementary Tables S7 and S8. Adjusted odds ratios (aORs) and 95% confidence intervals (CI) were calculated. Two-tailed *p* < 0.05 was considered statistically significant. Statistical analyses were performed using IBM SPSS Statistics version 31.0.0 (IBM Corp., Armonk, NY, USA), Python version 3.9.0, statsmodels version 0.14.6, pandas version 2.0.2, and Matplotlib version 3.5.1. Further details are provided in Supplementary File S2.

## 3. Results

### 3.1. Patient Characteristics

Of the 1423 enrolled patients, 372 (26.1%) patients received clazosentan. Of them, 341 patients (91.7%) completed the planned 14-day treatment and were included in the present analysis. Thirty-one patients (8.3%) discontinued clazosentan before day 14 and were therefore excluded. The reasons for premature discontinuation were pulmonary complications in 9 patients, cardiac complications in 8, hypotension in 4, cerebral edema in 4, deterioration of general condition in 3, intracranial hemorrhage in 2, and septic shock in 1. Among the 31 patients who discontinued clazosentan prematurely, 7 patients (22.6%) died before discharge. Clazosentan discontinuation was significantly more frequent among patients receiving adjunctive Fasudil than among those not receiving Fasudil (17/117 [14.5%] vs. 14/255 [5.5%], *p* = 0.007). However, none of the individual principal reasons for discontinuation differed significantly between the groups (Supplementary Table S1).

Finally, 341 received clazosentan with completion of 14 days and were included in the analysis (Figure 1). The median age was 64.0 (52.0–75.0) years, and 73.0% were female. The median WFNS Grade was II (I–IV), and the median Fisher CT group was 3 (3–3). Endovascular coiling was performed in 47.2% (161/341) of cases. Among these patients, 29.3% (100/341) used adjunctive Fasudil in addition to clazosentan during the VS phase, while 70.7% (241/341) received clazosentan alone (Table 1).

Compared with the clazosentan-alone group, the adjunctive Fasudil group had a lower proportion of women, a different distribution of WFNS grade and Fisher CT group, and higher frequencies of spinal and cisternal drainage. Other baseline characteristics were broadly similar between the groups.

Among patients with available outcome data, 17.6% (58/329) developed AVS, and 17.7% (58/327) developed cerebral infarction. Poor functional outcomes (mRS 3–6) were observed in 41.6% (142/341) at discharge and 25.2% (82/325) at 6 months. These outcomes did not differ significantly between the two groups (Table 2).

### 3.2. Factors Associated with AVS

In the univariable analysis, patients who developed AVS had a higher WFNS grade than those who did not. Diabetes mellitus, cilostazol use, and statin use were also associated with AVS. In the multivariable analysis, higher WFNS grade (aOR 1.39 [95% CI 1.12–1.74]) and the use of adjunctive Fasudil (aOR 2.41 [1.21–4.81]) were associated with higher odds of AVS (Table 3).

### 3.3. Factors Associated with Cerebral Infarction

In the univariable analysis, patients with cerebral infarction had a higher WFNS grade than those without cerebral infarction. Diabetes mellitus and statin use were also associated with cerebral infarction. In the multivariable analysis, higher WFNS grade (aOR 1.38 [1.12–1.70]) and adjunctive Fasudil use (aOR 2.10 [1.08–4.09]) were independently associated with cerebral infarction, whereas statin use was not (Table 4).

### 3.4. Factors Associated with Poor Functional Outcomes (mRS Score ≥ 3) at Discharge

In the univariable analysis, older age, history of stroke, higher WFNS grade, larger aneurysm size, higher Fisher CT group, ventricular drainage, cisternal drainage, statin use, AVS, cerebral infarction, cerebral complications, and systemic complications were associated with poor functional outcome at discharge. In the multivariable analysis, older age (aOR 1.08 [1.05–1.11]), higher WFNS grade (aOR 2.01 [1.58–2.55]), higher Fisher CT group (aOR 3.25 [1.48–7.13]), statin use (aOR 2.76 [1.27–6.03]), cerebral complications (aOR 3.03 [1.34–6.87]), and systemic complications (aOR 4.08 [1.62–10.24]) were independently associated with poor functional outcome at discharge. Adjunctive Fasudil (aOR 1.37 [0.62–3.03]) was not associated with poor functional outcome at discharge (Table 5).

### 3.5. Factors Associated with Poor Functional Outcomes (mRS Score ≥ 3) at 6 Months

In the univariable analysis, older age, higher WFNS grade, larger aneurysm size, higher Fisher CT group, ventricular drainage, cisternal drainage, statin use, AVS, cerebral infarction, cerebral complications, and systemic complications were associated with poor functional outcome at 6 months. In the multivariable analysis, older age (aOR 1.09 [1.05–1.14]), higher WFNS grade (aOR 2.78 [2.02–3.82]), adjunctive Fasudil use (aOR 2.88 [1.08–7.71]), statin use (aOR 4.85 [1.90–12.35]), and systemic complications (aOR 7.21 [2.39–21.74]) were independently associated with poor functional outcome at 6 months (Table 6).

### 3.6. Complete-Case Sensitivity Analyses

In the complete-case analysis, adjunctive Fasudil remained significantly associated with AVS (aOR 2.15, 95% CI 1.14–4.06; *p* = 0.018), consistent with the primary multiple-imputation analysis (Supplementary Table S2). Similarly, the association between adjunctive Fasudil and cerebral infarction was unchanged (aOR 2.10, 95% CI 1.08–4.09; *p* = 0.029) (Supplementary Table S3). For poor functional outcome at discharge, adjunctive Fasudil was not significantly associated with the outcome in the complete-case analysis (aOR 1.40, 95% CI 0.60–3.25; *p* = 0.436) (Supplementary Table S4), which was also consistent with the primary analysis. In contrast, for poor functional outcome at 6 months, the direction of the association remained positive, but the effect estimate was attenuated and was no longer statistically significant in the complete-case analysis (aOR 1.58, 95% CI 0.60–4.16; *p* = 0.357) (Supplementary Table S5), differing from the primary multiple-imputation analysis. The missing data were summarized in Supplementary Table S6.

### 3.7. Model Performance

In the complete-case models, the area under the curve was 0.721 for AVS, 0.681 for cerebral infarction, 0.894 for poor functional outcome at discharge, and 0.914 for poor functional outcome at 6 months. The Hosmer–Lemeshow goodness-of-fit test did not indicate significant lack of fit for any model (*p* = 0.713, 0.110, 0.441, and 0.991, respectively) (Supplementary Table S9).

### 3.8. Propensity Score Weighting Sensitivity Analysis

The propensity-score analysis included 298 patients with complete baseline covariate data, including 79 patients receiving adjunctive Fasudil and 219 patients not receiving Fasudil. After stabilized inverse probability of treatment weighting, covariate balance was substantially improved. Most baseline variables achieved an absolute standardized mean difference of <0.10, although small residual imbalances remained for posterior circulation aneurysm and aneurysm size (Supplementary Table S7).

In the weighted analyses, adjunctive Fasudil use was significantly associated with AVS (odds ratio [OR] 2.29, 95% CI 1.19–4.41; *p* = 0.013), cerebral infarction (OR 2.74, 95% CI 1.48–5.07; *p* = 0.001), poor functional outcome at discharge (OR 1.79, 95% CI 1.07–3.02; *p* = 0.028), and poor functional outcome at 6 months (OR 2.23, 95% CI 1.22–4.05; *p* = 0.009) (Supplementary Table S8).

### 3.9. Sensitivity Analysis Using mRS 4–6 as a Stringent Definition of Poor Functional Outcome

In additional sensitivity analyses using mRS 4–6 as a more stringent definition of poor functional outcome, adjunctive Fasudil use was not significantly associated with poor functional outcome at discharge (aOR 1.30, 95% CI 0.57–2.97; *p* = 0.533) or at 6 months (aOR 1.31, 95% CI 0.45–3.76; *p* = 0.618) in the pooled multivariable analyses across 10 imputed datasets.

## 4. Discussion

In this multi-center retrospective observational study of 341 patients with aSAH treated with clazosentan, adjunctive Fasudil use was not associated with improved VS-related or functional outcomes. In the adjusted analyses, adjunctive Fasudil use was associated with higher odds of AVS, cerebral infarction, and poor functional outcome at 6 months. These findings raise questions regarding the additional benefit of combining Fasudil with clazosentan in routine clinical practice.

### 4.1. Comparison with Previous Studies

Previous real-world studies have also compared clazosentan-based management with conventional Fasudil-based strategies. In the multicenter RECOVER study [19], clazosentan-based management was associated with lower incidences of angiographic vasospasm (15.4% vs. 33.7%) and vasospasm-related DCI (4.4% vs. 10.2%) and with better functional outcome at discharge (62.2% vs. 50.5%), compared with Fasudil-based management. Similarly, another study [23] reported that, compared with a conventional protocol based on Fasudil and cilostazol, a clazosentan-centered protocol was associated with lower incidences of moderate-to-severe angiographic vasospasm (25.9% vs. 44.0%), vasospasm-related symptomatic infarction (6.2% vs. 16.0%), and rescue therapy for DCI (16.1% vs. 34.0%), as well as a higher rate of favorable functional outcome at discharge (79.0% vs. 66.0%). Importantly, these studies evaluated clazosentan and Fasudil primarily as alternative prophylactic strategies, whereas the present study specifically addressed whether adding Fasudil provides incremental benefit in patients already receiving clazosentan.

Fasudil is sometimes co-administered with clazosentan for VS prevention [31], but previous randomized controlled trials of clazosentan did not evaluate concomitant Fasudil use during the VS phase [7,8,9,10,11]; therefore, the clinical value of combining Fasudil with clazosentan remains uncertain. Several real-world studies have reported concomitant Fasudil use during clazosentan treatment (Table 7) [13,16,18,25,28], but most focused on the safety profile of clazosentan, fluid retention, or treatment discontinuation rather than the incremental effect of Fasudil on VS-related and functional outcomes [13,16,25].

Among these reports, two are relevant to our study. Muraoka et al. provided one of the earliest real-world reports of clazosentan combination therapy after its introduction in Japan [18]. In that study, adjunctive Fasudil (38.3%, 17/47 patients) did not reduce the incidence of VS and was associated with numerically higher rates of VS (44.4% vs. 24.1%), VS-related delayed cerebral ischemia (16.7% vs. 3.4%), and unfavorable outcome at discharge (27.8% vs. 13.8%) than the non-Fasudil group. However, the sample size was small, and multivariable analysis was not performed.

In contrast, Sakata et al. reported a large post-marketing surveillance study of 2967 patients treated with clazosentan [28]. In that study, Fasudil was used in 16.9% of patients and Fasudil plus ozagrel in 5.5%. The rates of cerebral VS and VS-related cerebral infarction were similar between the Fasudil and non-Fasudil groups (17.9% vs. 19.1% and 6.9% vs. 7.2%, respectively). Also, adjunctive Fasudil use was not associated with VS-related morbidity or mortality in univariable analysis (unadjusted odds ratio 1.00 [0.75–1.33]).

The present study extends these previous observations by evaluating adjunctive Fasudil use in a multi-center cohort of 341 clazosentan-treated patients with aSAH using multivariable analyses. In the unadjusted group comparison, AVS, cerebral infarction, and functional outcomes did not differ significantly between the adjunctive Fasudil and clazosentan-alone groups. However, after adjustment for baseline severity and clinically relevant covariables by multivariable analysis, adjunctive Fasudil use was associated with higher odds of AVS, cerebral infarction, and poor functional outcome at 6 months. These findings are consistent with the unfavorable direction observed in the early report by Muraoka et al. [18] and raise questions regarding the additional benefit of combining Fasudil with clazosentan in routine clinical practice.

### 4.2. Possible Explanations for the Observed Associations Between Adjunctive Fasudil Use and Clinical Outcomes

Several explanations may account for the unfavorable association between adjunctive Fasudil and AVS, cerebral infarction, and poor functional outcome at 6 months. One possible explanation is that excessive systemic vasodilation caused by concomitant clazosentan and Fasudil may adversely affect systemic hemodynamics and, consequently, cerebral circulation during the VS phase. Clazosentan inhibits endothelin-A receptor-mediated vasoconstriction, whereas Fasudil induces vasodilation through Rho-kinase inhibition. Although this combination could theoretically provide additive protection against VS, it may also promote vasodilatory edema, fluid retention, hypotension, or hemodynamic instability. Consistent with this possibility, previous real-world studies have suggested that combined clazosentan and Fasudil therapy may be associated with fluid retention [13,18].

These systemic complications may, in turn, attenuate the expected benefit of VS prophylaxis. Sakata et al. reported that patients with fluid retention–related adverse drug reactions had a higher incidence of VS-related morbidity/mortality events [28]. These findings suggest that fluid retention and hemodynamic instability during the VS phase may worsen cerebral oxygen delivery, complicate fluid and blood pressure management, and thereby contribute to ischemic injury and delayed functional recovery.

However, residual confounding by indication remains an important alternative explanation. Adjunctive Fasudil may have been preferentially administered to patients perceived to be at higher risk for VS-related complications. Therefore, the present findings should not be interpreted as causal evidence that Fasudil directly worsens outcomes. Nevertheless, given the absence of a clear additive benefit and the unfavorable association observed after adjustment, routine concomitant use of Fasudil with clazosentan should be considered cautiously.

### 4.3. Limitations

First, the retrospective, observational nature of this study precludes definitively establishing causality. The decision to administer adjunctive Fasudil was left to the treating physicians’ discretion, rendering our findings highly susceptible to confounding by indication; physicians may have preferentially prescribed Fasudil to patients and perceived as having a higher baseline risk for VS. Because treatment allocation was not randomized, residual confounding cannot be completely eliminated despite multivariable adjustment and propensity score weighting. Therefore, the observed associations between adjunctive Fasudil and worse outcomes may reflect residual confounding by indication rather than a direct harmful effect of Fasudil.

Second, although multivariable adjustment and multiple imputation were performed, unmeasured confounders may have remained, including institutional treatment preferences, detailed hemodynamic management, fluid balance, cardiopulmonary and renal function, nutritional status [37,38,39], and the indication for adjunctive Fasudil administration.

Third, the sample size was modest, particularly in the adjunctive Fasudil group, which may have limited statistical power and precision of the estimates. Also, functional outcome after aSAH is multifactorial and may be influenced by factors other than VS-related complications, including initial hemorrhage severity, treatment-related complications, systemic complications, rehabilitation, and post-discharge care.

Fourth, because the present analysis included only patients who completed 14 days of clazosentan treatment, patients who discontinued clazosentan early because of adverse events or severe systemic complications were not included. Thirty-one of 372 clazosentan-treated patients discontinued treatment prematurely, mainly because of cardiopulmonary complications, hypotension, cerebral edema, or deterioration of general condition. This may have introduced selection bias and may limit the generalizability of the findings to all patients treated with clazosentan in routine practice.

Fifth, the association between adjunctive Fasudil use and poor 6-month functional outcome was not statistically significant in the complete-case sensitivity analysis, although the direction of the association was unchanged. This discrepancy may reflect the reduced sample size and wider confidence interval in the complete-case analysis, and the finding should therefore be interpreted cautiously.

Sixth, all participating centers were located in Japan, where pharmacological strategies for VS prevention and other aspects of aSAH management may differ from those commonly used in Europe and North America. Therefore, caution is warranted when generalizing our findings to other healthcare systems and treatment settings.

Finally, AVS was assessed according to local institutional protocols rather than a centralized core laboratory, which may have introduced interinstitutional variability. These findings should therefore be considered hypothesis-generating and require validation in prospective studies with standardized treatment protocols and prespecified criteria for adjunctive Fasudil use.

## 5. Conclusions

In this multi-center retrospective observational study of patients with aSAH treated with clazosentan, adjunctive Fasudil was not associated with improved VS-related or functional outcomes. Rather, adjunctive Fasudil use was associated with higher odds of AVS, cerebral infarction, and poor functional outcomes at 6 months. These findings raise questions regarding the additional benefit of combining Fasudil with clazosentan in routine clinical practice.

## Figures and Tables

**Table 1:** Clinical characteristics of patients with aneurysmal SAH treated with clazosentan ± Fasudil.

	Overall (*n* = 341)	Clazosentan + Fasudil Group (*n* = 100, 29.3%)	Clazosentan Group (*n* = 241, 70.7%)	*p* Value
Age (years), median (IQR)	64.0 (52.0–75.0)	63.5 (52.0–76.0)	64.0 (53.0–75.0)	0.892
Female sex (%)	249 (73.0%)	63 (63.0%)	186 (77.2%)	0.011 *
Past history				
Hypertension (%)	118/318 (37.1%)	36/88 (40.9%)	82/230 (35.7%)	0.437
Diabetes mellitus (%)	29/320 (9.1%)	12/89 (13.5%)	17/231 (7.4%)	0.126
Stroke (%)	14/320 (4.4%)	2/89 (2.2%)	12/231 (5.2%)	0.364
WFNS Grade, median (IQR)	II (I–IV)	II (I–IV)	II (I–IV)	0.016 *^†^
I (%)	114 (33.4%)	41 (41.0%)	73 (30.3%)	
II (%)	94 (27.6%)	28 (28.0%)	66 (27.4%)	
III (%)	17 (5.0%)	5 (5.0%)	12 (5.0%)	
IV (%)	46 (13.5%)	13 (13.0%)	33 (13.7%)	
V (%)	70 (20.5%)	13 (13.0%)	57 (23.7%)	
Aneurysm size (mm), mean (SD)	5.7 (2.8) (*n* = 323)	5.3 (2.1) (*n* = 91)	5.8 (3.0) (*n* = 232)	0.429
Aneurysm location				
ACA or ACoA (%)	131 (38.4%)	50 (50.0%)	81 (33.6%)	
ICA (%)	112 (32.9%)	28 (28.0%)	84 (34.9%)	
MCA (%)	71 (20.8%)	17 (17.0%)	54 (22.4%)	
VA (%)	27 (7.9%)	5 (5.0%)	22 (9.1%)	0.271 ^¶^
Fisher CT group, median (IQR)	3 (3–3); *n* = 339	3 (3–3)	3 (3–3); *n* = 239	0.604 ^†^
1 (%)	1/339 (0.3%)	1 (1.0%)	0/239 (0.0%)	
2 (%)	38/339 (11.2%)	10 (10.0%)	28/239 (11.7%)	
3 (%)	261/339 (77.0%)	80 (80.0%)	181/239 (75.7%)	
4 (%)	39/339 (11.5%)	9 (9.0%)	30/239 (12.6%)	0.003 *^§^
Surgical procedure				
Endovascular coiling, not surgical clipping (%endovascular coiling)	161 (47.2%)	52 (52.0%)	109 (45.2%)	0.284
Spinal drainage (%)	144 (42.2%)	52 (52.0%)	92 (38.2%)	0.022 *
Ventricular drainage (%)	111 (32.6%)	39 (39.0%)	72 (29.9%)	0.127
Cisternal drainage (%)	72 (21.1%)	34 (34.0%)	38 (15.8%)	<0.001 *
Cerebral vasospasm prophylaxis and other medications				
Cilostazol (%)	236 (69.2%)	63 (63.0%)	173 (71.8%)	0.123
Statin (%)	147 (43.1%)	50 (50.0%)	97 (40.2%)	0.118
Nicardipine (%)	3 (0.9%)	1 (1.0%)	2 (0.8%)	0.999

Abbreviations: ACA, anterior cerebral artery; ACoA, anterior communicating artery; CT, computed tomography; Fasudil, fasudil hydrochloride hydrate; ICA, internal carotid artery; IQR, interquartile range; MCA, middle cerebral artery; SAH, subarachnoid hemorrhage; SD, standard deviation; VA, vertebral artery; WFNS, World Federation of Neurosurgical Societies. *, *p* < 0.05. ^†^, treated as an ordinal variable, and tested by the Mann–Whitney U test. ^§^, treated as a three-categorical variable: 1 and 2, 3, or 4, and tested by the chi-square test. ^¶^, treated as a binomial variable, anterior circulation or posterior circulation, and tested by the chi-square test.

**Table 2:** Treatment outcomes of patients with SAH treated with clazosentan ± Fasudil.

	Overall (*n* = 341)	Clazosentan + Fasudil Group (*n* = 100, 29.3%)	Clazosentan Group (*n* = 241, 70.7%)	*p* Value
mRS score at discharge				0.877 ^†^
0 (%)	81 (23.8%)	31 (31.0%)	50 (20.7%)	
1 (%)	66 (19.4%)	13 (13.0%)	53 (22.0%)	
2 (%)	52 (15.2%)	15 (15.0%)	37 (15.4%)	
3 (%)	36 (10.6%)	13 (13.0%)	23 (9.5%)	
4 (%)	38 (11.1%)	13 (13.0%)	25 (10.4%)	
5 (%)	56 (16.4%)	12 (12.0%)	44 (18.3%)	
6 (%)	12 (3.5%)	3 (3.0%)	9 (3.7%)	
mRS score at 6 months (*n* = 325)				0.920 ^†^
0 (%)	161/325 (49.5%)	44/89 (49.5%)	117/236 (49.6%)	
1 (%)	54/325 (16.6%)	15/89 (16.9%)	39/236 (16.5%)	
2 (%)	28/325 (8.6%)	7/89 (7.9%)	21/236 (8.9%)	
3 (%)	15/325 (4.6%)	6/89 (6.7%)	9/236 (3.8%)	
4 (%)	14/325 (4.3%)	6/89 (6.7%)	8/236 (3.4%)	
5 (%)	36/325 (11.1%)	7/89 (7.9%)	29/236 (12.3%)	
6 (%)	17/325 (5.2%)	4/89 (4.5%)	13/236 (5.5%)	
Complications				
Angiographic vasospasm (%)	58/329 (17.6%)	24 (24.0%)	34/229 (14.8%)	0.058
IVR against cerebral vasospasm (%)	9 (2.6%)	5 (5.0%)	4 (1.7%)	0.130
Cerebral infarction (%)	58/327 (17.7%)	23 (23.0%)	35/227 (15.4%)	0.116
Cerebral complication (%)	92 (27.0%)	12 (12.0%)	80 (33.2%)	<0.001 *
Systemic complication (%)	75 (22.0%)	24 (24.0%)	51 (21.2%)	0.568

Abbreviations: IVR, interventional radiology; mRS, modified Rankin Scale; SAH, subarachnoid hemorrhage. *, *p* < 0.05. ^†^, treated as an ordinal variable, and tested by the Mann–Whitney U test.

**Table 3:** Multivariable logistic regression analysis of factors associated with angiographic vasospasm (*n* = 329).

	Univariable Analysis			Multivariable Analysis	
	Angiographic Vasospasm (−) (*n* = 271, 82.4%)	Angiographic Vasospasm (+) (*n* = 58, 17.6%)	*p* Values	aOR (95% CI)	*p* Values
Age (years; median, IQR)	64.0 (52.0–75.0)	62.0 (52.0–75.8)	0.472	0.99 (0.97–1.01)	0.406
Sex (%female)	199 (73.4%)	43 (74.1%)	1.000	1.41 (0.67–3.00)	0.366
Past history					
Hypertension (%)	95/254 (37.4%)	16/52 (30.8%)	0.430		
Diabetes mellitus (%)	29/256 (11.3%)	0/52 (0.0%)	0.007 **		†
Stroke (%)	9/256 (3.5%)	2/52 (3.8%)	1.000		
WFNS Grade, median (IQR)	II (I–IV)	III (II–IV)	0.009 **	1.39 (1.12–1.74)	0.003 **
Aneurysm size (mm), mean (SD)	5.7 (2.5)	6.3 (3.9)	0.855		
Aneurysm location; posterior circulation (%)	20 (7.4%)	6 (10.3%)	0.427		
Fisher CT group, median (IQR)	3 (3–3)	3 (3–3)	0.211	0.90 (0.45–1.80)	0.769
Surgical procedure					
Endovascular coiling, not surgical clipping (%endovascular coiling)	125 (46.1%)	30 (51.7%)	0.471		
Spinal drainage (%)	113 (41.7%)	28 (48.3%)	0.383		
Ventricular drainage (%)	88 (32.5%)	21 (36.2%)	0.645		
Cisternal drainage (%)	63 (23.2%)	9 (15.5%)	0.224		
Cerebral vasospasm prophylaxis and other medications					
Fasudil (%)	76 (28.0%)	24 (41.4%)	0.058	2.41 (1.21–4.81)	0.012 *
Cilostazol (%)	196 (72.3%)	31 (53.4%)	0.007 **	0.57 (0.28–1.17)	0.126
Statin (%)	128 (47.2%)	16 (27.6%)	0.008 **	0.51 (0.25–1.07)	0.073
Nicardipine (%)	2 (0.7%)	1 (1.7%)	0.442		
Outcomes and complications					
mRS score 0–2 at discharge (%)	168 (62.0%)	24 (41.4%)	0.005 **		‡
mRS score 0–2 at 6 months (%)	204/258 (79.1%)	29/55 (52.7%)	<0.001 ***		‡
IVR against cerebral vasospasm (%)	0 (0.0%)	9 (15.5%)	<0.001 ***		‡
Cerebral infarction (%)	27 (10.0%)	31/56 (55.4%)	<0.001 ***		‡
Cerebral complication (%)	57 (21.0%)	23 (39.7%)	0.004 **		‡
Systemic complication (%)	48 (17.7%)	15 (25.9%)	0.197		

In this multivariable analysis, we included the items that were significant in the univariable analysis, along with age, sex, Fisher computed tomography (CT) scale, and adjunctive Fasudil use. Abbreviations: CI, confidence interval; Fasudil, fasudil hydrochloride hydrate; IVR, interventional radiology; IQR, interquartile range; mRS, modified Rankin Scale; aOR, adjusted odds ratio; SD, standard deviation; WFNS, World Federation of Neurosurgical Societies; *, *p* < 0.05; **, *p* < 0.01; ***, *p* < 0.001; †, Variables with complete separation were excluded from the multivariable analysis. ‡, Blank cells indicate variables that were not included in the multivariable model because they occurred temporally after the outcome of interest and were therefore not considered appropriate explanatory variables.

**Table 4:** Multivariable logistic regression analysis of factors associated with cerebral infarction (*n* = 327).

	Univariable Analysis			Multivariable Analysis †	
	Cerebral Infarction (−) (*n* = 269, 82.3%)	Cerebral Infarction (+) (*n* = 58, 17.7%)	*p* Values	aOR (95% CI)	*p* Values
Age (years; median, IQR)	64.0 (52.0–75.0)	63.5 (52.0–76.8)	0.887	0.99 (0.97–1.01)	0.449
Sex (%female)	200 (74.3%)	40 (69.0%)	0.415	0.95 (0.48–1.87)	0.871
Past history					
Hypertension (%)	95/247 (38.5%)	16/57 (28.1%)	0.170		
Diabetes mellitus (%)	28/249 (11.2%)	1/57 (1.8%)	0.024 *	0.13 (0.02–1.00)	0.050
Stroke (%)	10/249 (4.0%)	1/57 (1.8%)	0.696		
WFNS Grade, median (IQR)	II (I–IV)	III (II–IV)	0.009 **	1.38 (1.12–1.70)	0.003 **
Aneurysm size (mm), mean (SD)	5.7 (2.6)	6.4 (3.6)	0.365		
Aneurysm location; posterior circulation (%)	22 (8.2%)	4 (6.9%)	1.000		
Fisher CT group, median (IQR)	3 (3–3)	3 (3–3)	0.800	0.76 (0.39–1.49)	0.423
Surgical procedure					
Endovascular coiling, not surgical clipping (%endovascular coiling)	123 (45.7%)	31 (53.4%)	0.312		
Spinal drainage (%)	116 (43.1%)	24 (41.4%)	0.884		
Ventricular drainage (%)	84 (31.2%)	25 (43.1%)	0.092		
Cisternal drainage (%)	59 (21.9%)	13 (22.4%)	1.000		
Cerebral vasospasm prophylaxis and other medications					
Fasudil (%)	77 (28.6%)	23 (39.7%)	0.116	2.10 (1.08–4.09)	0.029 *
Cilostazol (%)	185 (68.8%)	41 (70.7%)	0.876		
Statin (%)	111 (41.3%)	33 (56.9%)	0.041 *	1.50 (0.81–2.78)	0.197
Nicardipine (%)	2 (0.7%)	1 (1.7%)	0.444		
Outcomes and complications					
mRS score 0–2 at discharge (%)	171 (63.6%)	21/58 (36.2%)	<0.001 ***		‡
mRS score 0–2 at 6 months (%)	206/259 (79.5%)	27/52 (51.9%)	<0.001 ***		‡
Angiographic vasospasm (%)	25 (9.3%)	31 (53.4%)	<0.001 ***		‡
IVR against cerebral vasospasm (%)	2 (0.7%)	7 (12.1%)	<0.001 ***		‡
Cerebral complication (%)	51 (19.0%)	27 (46.6%)	<0.001 ***		‡
Systemic complication (%)	49 (18.2%)	12 (20.7%)	0.700		

Missing values were handled using 10 multiple imputations. In this multivariable analysis, we included the items that were significant in the univariable analysis, along with age, sex, Fisher computed tomography (CT) scale, and adjunctive Fasudil use. Abbreviations: CI, confidence interval; Fasudil, fasudil hydrochloride hydrate; IVR, interventional radiology; IQR, interquartile range; mRS, modified Rankin Scale; aOR, adjusted odds ratio; SD, standard deviation; WFNS, World Federation of Neurosurgical Societies; *, *p* < 0.05; **, *p* < 0.01; ***, *p* < 0.001; †, results are based on 10 multiple imputations for missing values. ‡, blank cells indicate variables that were not included in the multivariable model because they occurred temporally after the outcome of interest and were therefore not considered appropriate explanatory variables.

**Table 5:** Multivariable logistic regression analysis of factors associated with poor functional outcomes (mRS score ≥ 3) at discharge (*n* = 341).

	Univariable Analysis			Multivariable Analysis †	
	mRS Score 0–2 at Discharge (*n* = 199, 58.4%)	mRS Score 3–6 at Discharge (*n* = 142, 41.6%)	*p* Values	aOR (95% CI)	*p* Values
Age (years; median, IQR)	57.0 (50.0–68.0)	72.0 (61.3–79.0)	<0.001 ***	1.08 (1.05–1.11)	<0.001 ***
Sex (%female)	148 (74.4%)	101 (71.1%)	0.537	1.15 (0.51–2.56)	0.736
Past history					
Hypertension (%)	63/185 (34.1%)	55/133 (41.4%)	0.197		
Diabetes mellitus (%)	15/186 (8.1%)	14/134 (10.4%)	0.555		
Stroke (%)	3/186 (1.6%)	11/134 (8.2%)	0.005 **	3.38 (0.61–18.78)	0.163
WFNS Grade, median (IQR)	II (I–II)	IV (II–V)	<0.001 ***	2.01 (1.58–2.55)	<0.001 ***
Aneurysm size (mm), mean (SD)	5.4 (2.6)	6.5 (2.9)	0.002 **	1.02 (0.90–1.16)	0.734
Aneurysm location; posterior circulation (%)	14 (7.0%)	13 (9.2%)	0.543		
Fisher CT group, median (IQR)	3 (3–3)	3 (3–4); *n* = 140	<0.001 ***	3.25 (1.48–7.13)	0.003 **
Surgical procedure					
Endovascular coiling, not surgical clipping (%endovascular coiling)	102 (51.3%)	59 (41.5%)	0.080		
Spinal drainage (%)	84 (42.2%)	60 (42.3%)	1.000		
Ventricular drainage (%)	47 (23.6%)	64 (45.1%)	<0.001 ***	1.39 (0.59–3.26)	0.451
Cisternal drainage (%)	32 (16.1%)	40 (28.2%)	0.010 *	2.26 (0.82–6.19)	0.114
Cerebral vasospasm prophylaxis and other medications					
Fasudil (%)	59 (29.6%)	41 (28.9%)	0.904	1.37 (0.62–3.03)	0.435
Cilostazol (%)	140 (70.4%)	96 (67.6%)	0.635		
Statin (%)	75 (37.7%)	72 (50.7%)	0.020 *	2.76 (1.27–6.03)	0.011 *
Nicardipine (%)	2 (1.0%)	1 (0.7%)	1.000		
Outcomes and complications					
mRS score 0–2 at 6 months (%)	192/194 (99.0%)	51/131 (38.9%)	<0.001 ***		
Angiographic vasospasm (%)	24/192 (12.5%)	34/137 (24.8%)	0.005 **	1.51 (0.42–5.46)	0.527
IVR against cerebral vasospasm (%)	3 (1.5%)	5 (3.5%)	0.285		
Cerebral infarction (%)	21/192 (10.9%)	37/135 (27.4%)	<0.001 ***	1.86 (0.67–5.13)	0.233
Cerebral complication (%)	34 (17.1%)	58 (40.8%)	<0.001 ***	3.03 (1.34–6.87)	0.008 **
Systemic complication (%)	31 (15.6%)	44 (31.0%)	0.001 **	4.08 (1.62–10.24)	0.003 **

Missing values were handled using 10 multiple imputations. In this multivariable analysis, we included the items that were significant in the univariable analysis, along with age, sex, Fisher computed tomography (CT) scale, and adjunctive Fasudil use. Abbreviations: CI, confidence interval; Fasudil, fasudil hydrochloride hydrate; IVR, interventional radiology; IQR, interquartile range; mRS, modified Rankin Scale; aOR, adjusted odds ratio; SD, standard deviation; WFNS, World Federation of Neurosurgical Societies; *, *p* < 0.05; **, *p* < 0.01; ***, *p* < 0.001; †, results are based on 10 multiple imputations for missing values.

**Table 6:** Multivariable logistic regression analysis of factors associated with poor functional outcomes (mRS score ≥ 3) at 6 months (*n* = 325).

	Univariable Analysis			Multivariable Analysis †	
	mRS Score 0–2 at 6 Months (*n* = 243, 74.8%)	mRS Score 3–6 at 6 Months (*n* = 82, 25.2%)	*p* Values	aOR (95% CI)	*p* Values
Age (years; median, IQR)	61.0 (51.0–72.0)	72.0 (62.0–79.0)	<0.001 ***	1.09 (1.05–1.14)	<0.001 ***
Sex (%female)	181 (74.5%)	55 (67.1%)	0.200	0.52 (0.20–1.32)	0.169
Past history					
Hypertension (%)	79/223 (35.4%)	33/79 (41.8%)	0.344		
Diabetes mellitus (%)	20/224 (8.9%)	9/80 (11.2%)	0.515		
Stroke (%)	7/224 (3.1%)	5/80 (6.2%)	0.312		
WFNS Grade, median (IQR)	II (I–II)	IV (III–V)	<0.001 ***	2.78 (2.02–3.82)	<0.001 ***
Aneurysm size (mm), mean (SD)	5.5 (2.6)	6.5 (3.1)	<0.001 ***	1.02 (0.90–1.15)	0.734
Aneurysm location; posterior circulation (%)	16 (6.6%)	9(11.0%)	0.230		
Fisher CT group, median (IQR)	3 (3–3)	3 (3–4)	0.001 **	2.10 (0.77–5.76)	0.149
Surgical procedure					
Endovascular coiling, not surgical clipping (%endovascular coiling)	119 (49.0%)	35 (42.7%)	0.371		
Spinal drainage (%)	103 (42.4%)	34 (41.5%)	0.898		
Ventricular drainage (%)	62 (25.5%)	45 (54.9%)	<0.001 ***	1.56 (0.53–4.57)	0.414
Cisternal drainage (%)	39 (16.0%)	30 (36.6%)	<0.001 ***	3.41 (0.98–11.80)	0.053
Cerebral vasospasm prophylaxis and other medications					
Fasudil (%)	66 (27.2%)	23 (28.0%)	0.887	2.88 (1.08–7.71)	0.035 *
Cilostazol (%)	169 (69.5%)	54 (65.9%)	0.582		
Statin (%)	92 (37.9%)	46 (56.1%)	0.004 **	4.85 (1.90–12.35)	<0.001 ***
Nicardipine (%)	2 (0.8%)	1 (1.2%)	1.000		
Outcomes and complications					
Angiographic vasospasm (%)	29/233 (12.4%)	26/80 (32.5%)	<0.001 ***	1.07 (0.25–4.59)	0.928
IVR against cerebral vasospasm (%)	5 (2.1%)	3 (3.7%)	0.421		
Cerebral infarction (%)	27/233 (11.6%)	25/78 (32.1%)	<0.001 ***	1.17 (0.34–4.06)	0.809
Cerebral complication (%)	54 (22.2%)	35 (42.7%)	<0.001 ***	1.85 (0.71–4.79)	0.207
Systemic complication (%)	44 (18.1%)	30 (36.6%)	0.001 **	7.21 (2.39–21.74)	<0.001 ***

Missing values were handled using 10 multiple imputations. In this multivariable analysis, we included the items that were significant in the univariable analysis, along with age, sex, Fisher computed tomography (CT) scale, and adjunctive Fasudil use. Abbreviations: CI, confidence interval; Fasudil, fasudil hydrochloride hydrate; IVR, interventional radiology; IQR, interquartile range; mRS, modified Rankin Scale; aOR, adjusted odds ratio; SD, standard deviation; WFNS, World Federation of Neurosurgical Societies; *, *p* < 0.05; **, *p* < 0.01; ***, *p* < 0.001; †, results are based on 10 multiple imputations for missing values.

**Table 7:** Previous real-world reports on adjunctive Fasudil use in addition to clazosentan.

Author, Year	Number of Patients	Age	WFNS Grade	Fisher CT Group	Concomitant Prophylaxis	VS	Cerebral Infarction	Favorable Outcome (mRS 0–2) at Discharge	Favorable Outcome (mRS 0–2) at 6 Months	Main Findings Related to Fasudil
Muraoka et al., 2023 [18]	47	Mean 64.4 (SD 15.0)	I 23.4%, II 29.8%, III 19.1%, IV 12.8%, V 14.9%	1 (4.3%), 2 (17.0%), 3 (59.6%), 4 (19.1%)	Fasudil 38.3%, cilostazol 78.7%, ozagrel 31.9%, diuretic 42.6%	AVS: Overall 34.0%, Fasudil group 44.4%, Non-Fasudil group 24.1%.	Overall 8.5%, Fasudil group 16.7%, Non-Fasudil group 3.4%. †	Overall 61.7%, Fasudil group 61.1%, Non-Fasudil group 62.1%.	Not reported	Adjunctive Fasudil was associated with numerically higher VS, DCI, pulmonary edema, and unfavorable outcome.
Maeda et al., 2024 [16]	18	Median 74 [IQR 54.5–85.5]	I–III 66.7%, IV–V 33.3%	3 (83.3%)	Fasudil 100%	Symptomatic VS: Fasudil group 5.6%	Fasudil group 5.6%	Not reported	Fasudil group 61.1% at 3 months. ‡	In clazosentan + Fasudil group, fluid retention seemed more frequent.
Ando-Matsuoka et al., 2025 [13]	241 (Adverse Drug Event Report-based study)	75 [62.5–75]	Not described	Not described	Fasudil 36.9%, cilostazol 35.3%, ozagrel 18.3%, nicardipine 11.2%	Not reported	Not reported	Not reported	Not reported	Fasudil use was independently associated with clazosentan-induced fluid retention.
Sekimoto et al., 2025 [25]	32	57.9 (17.1)	I–III 50%, IV–V 50%	1 (3.1%), 2 (3.1%), 3 (84.4%), 4 (9.4%)	Fasudil 6.3%, cilostazol 43.8%, statin 31.3%	Not reported	Not reported	Not reported	Not reported	Fasudil was not related to fluid retention.
Sakata et al., 2026 [28]	2967 (post-marketing surveillance)	63.0 (14.4)	I 31.3%, II 28.1%, III 7.6%, IV 17.3%, V 15.6%	1 (2.4%), 2 (11.5%), 3 (74.3%), 4 (11.8%)	Fasudil 16.9%, ozagrel 7.1%, Fasudil and ozagrel 5.5%, neither Fasudil nor ozagrel 70.5%; Cilostazol 25.6%	Cerebral VS: Overall 18.8%, Fasudil group 17.9%, Non-Fasudil group 19.1%. ^¶^	Overall 7.1%, Fasudil group 6.9%, Non-Fasudil group 7.2%. ^¶^	Overall 80.2%. before 6 weeks/early discharge	Overall 75.0%	No clear differences were observed in cerebral VS, VS-related cerebral infarction, or VS-related morbidity/mortality events according to adjunctive Fasudil use.
Ours, 2026	341	64 [52–75]	I 33.4%, II 27.6%, III 5.0%, IV 13.5%, V 20.5%	1 (0.3%), 2 (11.2%), 3 (77.0%), 4 (11.5%)	Fasudil 29.3%, cilostazol 69.2%, statin 43.1%, nicardipine 0.9%	AVS: Overall 17.6%, Fasudil group 24.0%, Non-Fasudil group 14.8%.	Overall 17.7%, Fasudil group 23.0%, Non-Fasudil group 15.4%.	Overall 58.4%, Fasudil group 59.0%, Non-Fasudil group 58.1%.	Overall 74.8%, Fasudil group 74.3%, Non-Fasudil group 75.0%.	Adjunctive Fasudil use was associated with higher odds of AVS, cerebral infarction, and poor functional outcome at 6 months.

The studies presented are for descriptive, contextual comparison and do not constitute a systematic review. Abbreviations: AVS, angiographic vasospasm; CT, computed tomography; DCI, delayed cerebral ischemia; Fasudil, fasudil hydrochloride hydrate; IQR, interquartile range; mRS, modified Rankin Scale; SD, standard deviation; VS, vasospasm; WFNS, World Federation of Neurosurgical Societies. †, Cerebral infarction was reported as VS-related delayed cerebral ischemia. ‡, Good functional outcome was defined as an mRS score of 0–3 at 3 months. ^¶^, “Cerebral VS” refers to new cerebral VS assessed by the attending physician based on imaging findings, and “cerebral infarction” refers to VS-related cerebral infarction.

## Data Availability

De-identified data are available from the corresponding author upon reasonable request in accordance with the policy of the Shimane Prefectural Central Hospital Review Board.

## Supplementary Materials

The following supporting information can be downloaded at: https://www.mdpi.com/article/10.3390/jcm15166424/s1, Supplementary File S1: Details about the study protocol of DCI Japan. Supplementary File S2: Detailed methods. Supplementary Table S1: Principal reasons for premature clazosentan discontinuation according to adjunctive Fasudil use among 372 patients who initiated clazosentan. Supplementary Table S2: Complete-case multivariable logistic regression analyses for angiographic vasospasm. Supplementary Table S3: Complete-case multivariable logistic regression analyses for cerebral infarction. Supplementary Table S4: Complete-case multivariable logistic regression analyses for poor functional outcomes (mRS score ≥ 3) at discharge. Supplementary Table S5: Complete-case multivariable logistic regression analyses for poor functional outcomes (mRS score ≥ 3) at 6 months. Supplementary Table S6: Missing data. Supplementary Table S7: Baseline covariate balance before and after stabilized inverse probability of treatment weighting (IPTW). Supplementary Table S8: Stabilized IPTW analyses of clinical outcomes. Supplementary Table S9: Model performance.

## Abbreviations

The following abbreviations are used in this manuscript:

ACA	anterior cerebral artery
ACoA	anterior communicating artery
aOR	adjusted odds ratio
aSAH	aneurysmal subarachnoid hemorrhage
AVS	angiographic vasospasm
CI	confidence interval
CT	computed tomography
DCI	delayed cerebral ischemia
DCI Japan	Database of Cohort Study for Outcome of SAH in Japan
Fasudil	fasudil hydrochloride hydrate
ICA	internal carotid artery
IPTW	inverse probability of treatment weighting
IVR	interventional radiology
IQR	interquartile range
MCA	middle cerebral artery
mRS	modified Rankin Scale
OR	odds ratio
Q1–Q3	25th–75th percentile
RCT	randomized controlled trial
SAH	subarachnoid hemorrhage
SD	standard deviation
VA	vertebral artery
VS	vasospasm
WFNS	World Federation of Neurosurgical Societies
