# Supplementary material for: Adjunctive Fasudil Use and Outcomes After Clazosentan Treatment for Aneurysmal Subarachnoid Hemorrhage: A Multicenter DCI Japan Registry Study
Source: J Clin Med. 2026 Aug 19;15(16):6424. doi: 10.3390/jcm15166424 (PMC13514307; doi:10.3390/jcm15166424)
Supplement: Supplementary file 1 [file jcm-15-06424-s001.zip › Supplementary File S2 METHODS.pdf]

Supplementary File S2 SUPPLEMENTARY METHODS

2.4 Consistent treatment protocol of general management for aneurysmal SAH

Treatment protocol for aneurysmal SAH (aSAH) across all institutions followed the Japanese Guidelines for the Management of Stroke 2021.[29] Preoperative management included nicardipine to maintain normovolemia and a systolic blood pressure <130 mmHg. Surgical indications followed the Japanese Guidelines for the Management of Stroke 2021: patients with World Federation of Neurosurgical Societies (WFNS) Grades I–III were eligible for aneurysm treatment, while those with Grades IV and V were generally excluded unless they were young, middle-aged, or had a significant hematoma or hydrocephalus. Definitive treatment (surgical clipping or endovascular coiling) was performed within 4 days of onset by certified neurosurgeons following a thorough discussion. Postoperative care included prevention of vasospasm (VS), rehabilitation, and management of systemic complications. Symptomatic VS (SVS) was treated with intra-arterial Fasudil or percutaneous transluminal angioplasty as needed.

2.5 Inclusion and exclusion criteria

A flow diagram of patient selection is shown in **Figure 1**. This study included patients with aSAH who underwent surgical clipping or endovascular coil embolization for a ruptured aneurysm within 4 days of symptom onset. Exclusion criteria were: failure to identify a ruptured aneurysm on imaging; non-saccular aneurysms (e.g., dissecting, fusiform, infectious, or traumatic); prehospital modified Rankin Scale (mRS) ≥2 or unavailable; absence of definitive treatment; treatments other than simple surgical clipping or coiling (e.g., wrapping, trap and bypass, parent artery occlusion, decompression, or stent-assisted coiling); complex or crossover treatments; missing or inaccurate onset or treatment dates; or treatment initiated ≥4 days after onset. Among patients meeting these criteria, those who received clazosentan and completed 14 days of treatment after definitive treatment were included in the final analysis. Patients who discontinued clazosentan before day 14 were excluded because the present study was designed to evaluate outcomes associated with adjunctive Fasudil during a completed course of clazosentan prophylaxis.

2.6 Definition of VS

Cerebral VS was assessed using computed tomography (CT) angiography or digital subtraction angiography according to local protocols, typically immediately after surgery, on postoperative days 1, 7, and 14, and when new neurological deficits emerged. The classification into angiographic VS (AVS), SVS, and cerebral infarction followed the conventional model of vasoconstriction leading to ischemia.[30]

AVS was defined as ≥50% narrowing in any cerebral artery on angiography compared with preoperative baseline, excluding changes caused by atherosclerosis, catheter-induced spasm, or vessel hypoplasia. Diagnosis was based solely on imaging.[31]

SVS was defined as neurological deficits occurring during the VS phase (typically days 3–14 after aSAH onset); the appearance of focal neurological signs, or a ≥2-point decrease in Glasgow Coma Scale sustained for at least 1 hour, or both, was considered indicative of ischemia attributable to VS after excluding other potential causes of worsening (e.g., hydrocephalus, seizures, metabolic derangement, infection, or oversedation).[31]

Cerebral infarction was defined as new infarcts occurring within 6 weeks of SAH on CT, magnetic resonance imaging, or autopsy, which were absent on early postoperative imaging and not attributable to surgery, catheters, or hematoma. Infarcts from non-VS mechanisms (e.g., cardioembolism, atherothrombosis) were also included.

2.8 Variables and outcomes

Collected data included age, sex, history of hypertension, diabetes mellitus, stroke, World Federation of Neurosurgical Societies (WFNS) Grade, aneurysm size and location, Fisher CT group, treatment modality (surgical clipping or endovascular coiling), periprocedural management, and VS prophylaxis during the VS phase, including spinal/ventricular/cisternal drains, clazosentan completion, concomitant use of Fasudil, cilostazol, statins, and nicardipine. Radiological interventions for VS included intra-arterial Fasudil and angioplasty. Cerebral complications (hemorrhage, edema, infarction, infection) and systemic complications (infections requiring antibiotics, syndrome of inappropriate secretion of antidiuretic hormone, lung edema, arrhythmia) were documented.

The primary outcome was the occurrence of AVS. Secondary outcomes included cerebral infarction, good (mRS 0-2) or poor (mRS 3-6) functional outcome at discharge, and those at 6 months. We compared these outcomes between patients treated with clazosentan plus Fasudil and those treated with clazosentan alone. These associations were evaluated using the multivariable analyses described below.

In addition, sensitivity analyses were performed using mRS 4–6 as a more stringent definition of poor functional outcome at discharge and at 6 months, using the same multivariable models and multiple-imputation procedure as in the corresponding primary analyses.

2.9 Statistical analyses

Continuous variables with normal distribution were described as means (standard deviation [SD]) and compared using a t-test. Continuous variables that were not normally distributed and ordinal variables were described as medians [25th–75th percentile; Q1–Q3] and compared using the Mann-Whitney U test. Categorical variables were described as actual numbers and percentages, and the proportions were compared using chi-square or Fisher's exact tests. Significant variables from univariable analyses were included in the multivariable analysis, along with age (≥76 years), sex, WFNS Grade (Grade V vs I–IV), and Fisher CT group (1–2, 3, or 4). These categorical variables were included to evaluate the influence of underrepresented patient subgroups in the Japanese phase 3 RCT.[10] Given the exploratory nature of this real-world observational study and the lack of prior data on clazosentan discontinuation, no statistical power calculation was conducted before the study, and the sample size was based on available data.

A multivariable logistic regression model was used to investigate the association between each outcome and the factors described above. To prevent overfitting, given the limited number of events, the number of covariates included in each multivariable model was determined solely by clinical relevance and event frequency.

Missing data were handled using multiple imputation in IBM SPSS Statistics version 31.0.0 (IBM Corp., Armonk, NY, USA). Ten imputed datasets were generated using the entire analytic cohort; imputation was not performed separately according to adjunctive Fasudil use. The imputation model included the Fasudil treatment indicator and variables used in the corresponding analyses. Variables without missing values were retained as predictors but were not themselves imputed. Each regression model was fitted separately to the 10 imputed datasets, and parameter estimates and standard errors were pooled using Rubin's rules. Outcome variables were not imputed. The pooled analyses were regarded as the primary analyses; Complete-case analyses were performed as sensitivity analyses and are presented in the **Supplementary Tables 2-5**.

Because of an imbalance in outcome frequencies, Firth's penalized likelihood method was applied. If there were variables with a correlation

coefficient of 0.8 or higher and suggested high multicollinearity, one was selected for use based on clinical reasonability. Odds ratios (ORs) and 95% confidence intervals (CIs) of items were determined. Statistical significance was set at a two-tailed $p < 0.05$. SPSS Statistics version 31.0.0 (IBM Corp., Armonk, NY, USA), Python 3.9.0, Statsmodels 0.14.6, Pandas 2.0.2, and Matplotlib 3.5.1 were used.

2.10 Model performance assessment

Model discrimination was assessed using the area under the receiver operating characteristic curve (AUC), and goodness-of-fit was evaluated using the Hosmer–Lemeshow test. Because the primary analyses were based on multiple imputation, these model-performance assessments were performed using the corresponding complete-case datasets with the same covariate specifications as the multivariable sensitivity models.

2.11 Propensity score weighting analysis

As a sensitivity analysis addressing confounding by indication, we performed inverse probability of treatment weighting based on the propensity score for adjunctive Fasudil use. The propensity score was estimated using a logistic regression model that included baseline variables considered clinically relevant to treatment selection: age, sex, WFNS grade, hypertension, diabetes mellitus, previous stroke, posterior circulation aneurysm, aneurysm size, Fisher CT group, and treatment modality (endovascular coiling versus surgical clipping). Stabilized inverse probability-of-treatment weights were calculated from the estimated propensity scores. Covariate balance before and after weighting was assessed using standardized mean differences, with an absolute standardized mean difference of <0.10 considered indicative of adequate balance.

After weighting, weighted univariable logistic regression models with robust standard errors were used to evaluate the associations between adjunctive Fasudil use and AVS, cerebral infarction, poor functional outcome at discharge, and poor functional outcome at 6 months. Adjunctive Fasudil use was entered as the sole explanatory variable in each weighted outcome model.
