# Supplementary material for: Adjunctive Fasudil Use and Outcomes After Clazosentan Treatment for Aneurysmal Subarachnoid Hemorrhage: A Multicenter DCI Japan Registry Study
Source: J Clin Med. 2026 Aug 19;15(16):6424. doi: 10.3390/jcm15166424 (PMC13514307; doi:10.3390/jcm15166424)
Supplement: Supplementary file 1 [file jcm-15-06424-s001.zip › Supplementary Tables.pdf]

Supplementary Table S1. Principal reasons for premature clazosentan discontinuation according to adjunctive Fasudil use among 372 patients who initiated clazosentan

**Event**	**Overall, n=372**	**Clazosentan + Fasudil group, n=117 (31.5%)**	**Clazosentan group, n=255 (68.5%)**	**p value**
Premature clazosentan discontinuation	31 (8.3%)	17 (14.5%)	14 (5.5%)	0.007
Pulmonary complication	9 (2.4%)	5 (4.3%)	4 (1.6%)	0.147
Cardiac complication	8 (2.2%)	5 (4.3%)	3 (1.2%)	0.115
Hypotension	4 (1.1%)	3 (2.6%)	1 (0.4%)	0.094
Cerebral edema	4 (1.1%)	1 (0.9%)	3 (1.2%)	1.000
Deterioration of general condition	3 (0.8%)	2 (1.7%)	1 (0.4%)	0.234
Intracranial hemorrhage	2 (0.5%)	1 (0.9%)	1 (0.4%)	0.531
Septic shock	1 (0.3%)	0 (0.0%)	1 (0.4%)	1.000

The listed complications represent the principal reasons for premature discontinuation of clazosentan. Each patient was classified according to one principal reason; therefore, the categories were mutually exclusive. Complications that occurred during the general clinical course and did not result in discontinuation of clazosentan were not included.

Supplementary Table S2. Complete-case multivariable logistic regression analyses for angiographic vasospasm

Variable	aOR (95% CI)	p value
Age	0.99 (0.97–1.01)	0.241
Female sex	1.27 (0.63–2.55)	0.504
WFNS grade	1.36 (1.11–1.67)	0.003*
Fisher CT group	1.03 (0.53–2.02)	0.928
Adjunctive Fasudil	2.15 (1.14–4.06)	0.018*
Cilostazol	0.49 (0.25–0.99)	0.046*
Statin	0.57 (0.28–1.19)	0.133

This is a sensitivity analysis for **Table 3**, using complete case (n=329, outcome=58, 17.6%). "Diabetes mellitus" was excluded because of complete separation.
Abbreviations: CI, confidence interval; CT, computed tomography; Fasudil, fasudil hydrochloride hydrate; aOR, adjusted odds ratio; WFNS, World Federation of Neurosurgical Societies; *, p<0.05

Supplementary Table S3. Complete-case multivariable logistic regression analyses for cerebral infarction

Variable	aOR (95% CI)	p value
Age	0.99 (0.97–1.01)	0.449
Female sex	0.95 (0.48–1.87)	0.871
Diabetes mellitus	0.13 (0.02–1.00)	0.050
WFNS grade	1.38 (1.12–1.70)	0.003*
Fisher CT group	0.76 (0.39–1.49)	0.423
Adjunctive Fasudil	2.10 (1.08–4.09)	0.029*
Statin	1.50 (0.81–2.78)	0.197

This is a sensitivity analysis for Table 4, using complete case analysis (n=306, outcome=57, 18.6%).
Abbreviations: CI, confidence interval; CT, computed tomography; Fasudil, fasudil hydrochloride hydrate; aOR, adjusted odds ratio; WFNS, World Federation of Neurosurgical Societies; *, p<0.05

Supplementary Table S4. Complete-case multivariable logistic regression analyses for poor functional outcomes (mRS score ≥ 3) at discharge

Variable	aOR (95% CI)	p value
Age	1.08 (1.05–1.11)	<0.001*
Female sex	1.25 (0.56–2.80)	0.591
Previous stroke	1.79 (0.28–11.40)	0.536
WFNS grade	2.00 (1.56–2.57)	<0.001*
Aneurysm size	1.02 (0.90–1.15)	0.734
Fisher CT group	3.09 (1.40–6.82)	0.005*
Ventricular drainage	1.32 (0.54–3.23)	0.535
Cisternal drainage	2.13 (0.75–6.06)	0.154
Adjunctive Fasudil	1.40 (0.60–3.25)	0.436
Statin	2.72 (1.24–5.95)	0.012*
Angiographic vasospasm	1.92 (0.63–5.90)	0.254
Cerebral infarction	1.89 (0.70–5.12)	0.208
Cerebral complication	2.53 (1.07–5.98)	0.034*

Systemic complication	3.86 (1.50–9.93)	0.005*

This is a sensitivity analysis for **Table 5**, using complete case (n=288, outcome=118, 40.9%). Abbreviations: CI, confidence interval; CT, computed tomography; Fasudil, fasudil hydrochloride hydrate; mRS, modified Rankin Scale; aOR, adjusted odds ratio; WFNS, World Federation of Neurosurgical Societies; *, p<0.05

Supplementary Table S5. Complete-case multivariable logistic regression analyses for poor functional outcomes (mRS score ≥ 3) at 6 months.

Variable	aOR (95% CI)	p value
Age	1.08 (1.04–1.12)	<0.001*
Female sex	0.88 (0.37–2.12)	0.783
WFNS grade	2.28 (1.70–3.06)	<0.001*
Aneurysm size	1.03 (0.90–1.17)	0.677
Fisher CT group	2.32 (0.94–5.77)	0.069
Ventricular drainage	1.10 (0.40–3.04)	0.847
Cisternal drainage	3.38 (1.05–10.81)	0.040*
Adjunctive Fasudil	1.58 (0.60–4.16)	0.357
Statin	4.83 (1.88–12.42)	0.001*
Angiographic vasospasm	2.84 (0.94–8.58)	0.064
Cerebral infarction	2.03 (0.70–5.87)	0.190
Cerebral complication	1.27 (0.47–3.48)	0.638
Systemic complication	4.03 (1.45–11.22)	0.008*

This is a sensitivity analysis for **Table 6**, using complete case (n=293, outcome=69, 23.5%). Abbreviations: CI, confidence interval; CT, computed tomography; Fasudil, fasudil hydrochloride hydrate; mRS, modified Rankin Scale; aOR, adjusted odds ratio; WFNS, World Federation of Neurosurgical Societies; *, p<0.05

Supplementary Table S6. Missing data

	Overall (n=341)	Clazosentan + Fasudil group (n=100, 29.3%)	Clazosentan group (n=241, 70.7%)
Variables	Missing number (%)	Missing number (%)	Missing number (%)
Hypertension	23 (6.7%)	12 (12.0%)	11 (4.6%)
Diabetes mellitus	21 (6.2%)	11 (11.0%)	10 (4.1%)
Previous stroke	21 (6.2%)	11 (11.0%)	10 (4.1%)
Aneurysm size	18 (5.3%)	9 (9.0%)	9 (3.7%)
Fisher CT group	2 (0.6%)	0 (0.0%)	2 (0.8%)
Angiographic vasospasm	12 (3.5%)	0 (0.0%)	12 (5.0%)
Symptomatic vasospasm	17 (5.0%)	2 (2.0%)	15 (6.2%)
Cerebral infarction	14 (4.1%)	0 (0.0%)	14 (5.8%)
6-month mRS	16 (4.7%)	11 (11.0%)	5 (2.1%)

Abbreviations: CT, computed tomography; Fasudil, fasudil hydrochloride hydrate; mRS, modified Rankin Scale

Supplementary Table S7. Baseline covariate balance before and after stabilized IPTW

Covariate	Before weighting		SMD before weighting	After weighting		SMD after weighting
	Fasudil (n=79)	No Fasudil (n=219)		Fasudil	No Fasudil	
Age, years	63.70 (15.26)	62.81 (14.26)	0.060	64.51 (15.31)	63.15 (13.96)	0.093*
Female sex	58.2%	78.5%	-0.448	74.9%	73.8%	0.024*
WFNS grade	2.14 (1.34)	2.77 (1.58)	-0.431	2.66 (1.49)	2.61 (1.55)	0.032*
Hypertension	40.5%	34.2%	0.130	35.5%	35.2%	0.007*
Diabetes mellitus	15.2%	7.3%	0.251	9.1%	9.7%	-0.020*
Previous stroke	1.3%	4.6%	-0.197	4.8%	3.7%	0.053*
Posterior circulation aneurysm	3.8%	7.8%	-0.171	3.6%	6.6%	-0.137
Aneurysm size, mm	5.23 (2.06)	5.79 (3.00)	-0.220	5.28 (2.08)	5.61 (2.84)	-0.134
Fisher CT group	2.95 (0.50)	3.01 (0.50)	-0.128	2.99 (0.54)	2.99 (0.51)	-0.010*
Endovascular coiling	46.8%	45.7%	0.024	43.9%	46.1%	-0.045*

Continuous variables are presented as mean (standard deviation), and categorical variables as percentages. Weighted values are estimates after stabilized inverse probability of treatment weighting. An absolute standardized mean difference (SMD) <0.10 was considered indicative of adequate balance (*). Small residual imbalances remained for posterior circulation aneurysm and aneurysm size.

Abbreviations: CT, computed tomography; Fasudil, fasudil hydrochloride hydrate; IPTW, inverse probability-of-treatment weights; WFNS, World Federation of Neurosurgical Societies;

Supplementary Table S8. Stabilized IPTW analyses of clinical outcomes

Outcome	Analyzed number	Events, number (%)	Weighted risk: Fasudil (+)	Weighted risk: Fasudil (-)	OR (95% CI)	p value
Angiographic vasospasm	288	42 (14.6%)	24.9%	12.7%	2.29 (1.19–4.41)	0.013*
Cerebral infarction	286	52 (18.2%)	31.7%	14.5%	2.74 (1.48–5.07)	0.001*
Poor functional outcome at discharge (mRS 3–6)	298	122 (40.9%)	53.2%	38.8%	1.79 (1.07–3.02)	0.028*
Poor functional outcome at 6 months (mRS 3–6)	282	70 (24.8%)	38.7%	22.1%	2.23 (1.22–4.05)	0.009*

Abbreviations: CI, confidence interval; IPTW, inverse probability-of-treatment weighting; mRS, modified Rankin Scale; OR, odds ratio.

Supplementary Table S9. Model performance

Outcome	Analyzed number	Events, number (%)	AUC	Hosmer-Lemeshow p
Angiographic vasospasm	329	58	0.721	0.713
Cerebral infarction	306	57	0.681	0.110
Poor functional outcome at discharge (mRS 3–6)	288	118	0.894	0.441
Poor functional outcome at 6 months (mRS 3–6)	293	69	0.914	0.991

Abbreviations: AUC, area under the curve; mRS, modified Rankin Scale.
